# Practical guide for the diagnosis and treatment of localized and generalized cutaneous pruritus (chronic itch with no underlying pruritic dermatosis)

**DOI:** 10.1111/1346-8138.17565

**Published:** 2024-12-12

**Authors:** Takashi Hashimoto, Satoshi Okuno

**Affiliations:** ^1^ Department of Dermatology National Defense Medical College Tokorozawa Japan

**Keywords:** cutaneous pruritus, generalized pruritus, itch, localized pruritus, neuropathic itch

## Abstract

Itch, also known as pruritus, is one of the most prevalent symptoms observed in dermatological practices. Itch frequently arises from primary pruritic dermatoses, although it may also manifest in the absence of a primary pruritic skin rash. The latter itchy condition is referred to as “cutaneous pruritus” in the Japanese guidelines published in 2020. Cutaneous pruritus can be classified into two categories based on its distribution: localized cutaneous pruritus and generalized cutaneous pruritus. Localized cutaneous pruritus is indicative of a neuropathic cause, whereas generalized cutaneous pruritus suggests underlying systemic disease(s), drug‐induced itch, psychogenic itch (also known as functional itch disorder), or chronic pruritus of unknown origin (CPUO). Systemic diseases associated with cutaneous pruritus include disorders of iron metabolism, chronic kidney disease, chronic liver disease (especially cholestasis), endocrine/metabolic diseases, hematological disorders, and malignant solid tumors. CPUO is a term used to describe chronic itch that is often generalized and for which no underlying cause can be identified despite a comprehensive and careful diagnostic workup. A variety of treatment approaches are available for cutaneous pruritus, including device‐based physical therapies (such as phototherapy) and medications that act on the itch‐perception processing pathway from the skin, peripheral sensory nerves, the spinal cord, to the brain. This review presents an overview of the current knowledge regarding cutaneous pruritus, from its underlying pathophysiologic mechanisms to the diagnostic procedures and treatment approaches that are currently available.

## 1 INTRODUCTION

Itch, also known as pruritus, is defined as an uncomfortable sensation that provokes an urge to scratch.^1^ It impairs the quality of life (QOL) of those experiencing itch and can be debilitating and even lethal when it becomes severe and/or chronic.^2^ Therefore, dermatologists and physicians who see patients with itch must be knowledgeable about it. This review will provide an overview of the pathophysiology and treatment strategies for itch, with a particular focus on itch with no underlying primary pruritic skin disease.

## 2 TERMINOLOGY AND CLASSIFICATION OF ITCH

Various classifications of itch have been proposed based on such criterion as the duration of the condition, the underlying pathogenesis, the causes, the affected areas, and its characteristics (Table 1).

**TABLE 1:** Classifications of itch.

Classification	Type of itch	Definition
Duration	Acute itch	Itch lasting for less than 6 weeks
	Chronic itch	Itch lasting for 6 weeks or more
Trigger	Chemical itch	Itch induced by chemical substances that elicit itch (pruritogens)
	Mechanical itch	Itch evoked by mechanical stimuli
Pruritogen	Histaminergic itch	Itch induced by histamine
	Nonhistaminergic itch	Itch induced by a pruritogen other than histamine
Cause	Inflammatory itch	Itch associated with inflammatory condition
	Neuropathic itch	Itch with neuropathic cause
Affected area	Generalized itch	Itch that is widespread across the body and not limited to a specific body area
	Localized itch	Itch that is fixed and localized to specific body part(s)

### 2.1 Acute itch and chronic itch

Itch that persists for a duration of fewer than 6 weeks is classified as acute itch. Chronic itch is itch that lasts for 6 weeks and more.

### 2.2 Chemical itch and mechanical itch

The majority of itching sensation occurs when sensory nerve fibers are activated by a single or combination of substances known as pruritogens (Table 2). Furthermore, innocuous mechanical force can elicit itching sensation, particularly in patients with chronic itch. The former type of itch is referred to as chemical itch and the latter is designated as mechanical itch.

**TABLE 2:** Pruritogens and their receptors.

Category	Pruritogens	Receptors
Amines	Histamine	H1 receptor
	Serotonin	5‐HT2 receptor
Proteases	Kallikreins	PAR‐2
	Tryptase	
	Trypsin	
	Cathepsin S	
	Exogenous proteases	
Neuropeptides	substance P	NK1R and MrgprX2
	Endothelin‐1	ETA
	Opioids	MORs/KORs
Lipid mediators	PAF	PAF receptor
	LPA	LPA5 receptor
Cytokines	IL‐4/13	IL‐4Rα
	IL‐31	IL‐31RA and oncostatin M receptor
Mrgpr agonists	Chloroquine	MrgprX
	Bilirubin	MrgprX4
	Bile acids	MrgprX4

Abbreviations: ETA, endothelin receptor type A; IL, interleukin; KOR, kappa‐opioid receptor; LPA, lysophosphatidic acid; MOR, mu opioid receptor; MrgprX, Mas‐related G‐protein coupled receptor X; NK1R, neurokinin 1 receptor; PAF, platelet‐activating factor; PAR‐2, protease‐activated receptor 2.

### 2.3 Histaminergic itch and nonhistaminergic itch

Itch‐conveying sensory nerve fibers in the skin can be divided into two subsets: histaminergic nerves and nonhistaminergic nerves. Histaminergic nerves express histamine receptor H1R and are responsible for histamine‐induced itch. Nonhistaminergic nerves show responsiveness to pruritogens other than histamine, leading to nonhistaminergic itch.

### 2.4 Inflammatory itch and neuropathic itch

Itching sensation is usually associated with inflammatory conditions, particularly allergic inflammation (e.g., atopic dermatitis, urticaria, and contact dermatitis). The presence of inflammation facilitates the secretion of pruritogen from various types of cells. Secreted pruritogens then act on sensory nerve fibers, resulting in itching. This type of itch is referred to as inflammatory itch. On the other hand, some types of itching sensation do not necessarily arise from this inflammation‐pruritogen axis. Itch‐conveying nerve fibers may autonomically generate electrical impulses for some reasons (e.g., damage, inflammation, degeneration, and/or dysfunction of nerve fibers) even in the absence of apparent inflammation, resulting in itching on their innervated site(s). This itch is called neuropathic itch.^3^

### 2.5 Generalized itch and localized itch

The term generalized itch is used to describe the presence of itching sensation across the body. Localized itch represents fixed itch localized to the specific part of the body.

### 2.6 Alloknesis and hyperknesis

Patients with chronic itch often show sensitivity to itch stimuli. This itch sensitivity phenomenon can be classified into two categories: alloknesis and hyperknesis. Alloknesis is defined as considerable itch evoked by normal or nonpruritic stimuli, such as clothing. Hyperknesis is increased itch perceived in response to normal itch‐evoking stimuli.^4^, ^5^

## 3 ITCH ASSESSMENT TOOLS

Itch is a subjective and multidimensional experience, rendering it challenging to quantify. Moreover, there is currently a dearth of reliable biomarkers or objective tools for evaluating the severity of itch. Consequently, itch should be assessed based primarily on verbal expressions provided by patients. There are several major assessment tools for itch (Table 3).^6^, ^7^

**TABLE 3:** Itch assessment tools.

Numerical rating scale (NRS)	An 11‐point Likert score from zero (no itch) to 10 (the worst itch imaginable)
Visual analogue scale (VAS)	A straight line with one end meaning no itch and the other end meaning the worst itch imaginable, on which a patient marks a point that matches his/her itch
Verbal rating scale (VRS)	A five‐point Likert score from zero (no itch) to four (very severe itch)
5‐D itch scale	A brief questionnaire with five dimensions: duration, degree, direction, disability, and distribution
Others	Including Dermatology Life Quality Index (DLQI) and ItchyQoL

### 3.1 Numerical rating scale

Patients are asked to rate their itch on an 11‐point Likert score from 0 to 10, with 0 indicating no itch and 10 the worst itch imaginable. The score for itch intensity is provided out of 10, e.g., 7 of 10, or just 7/10. The peak pruritus numerical rating scale (PP‐NRS), also known as the worst itch numerical rating scale (WI‐NRS), represents the individual's worst itch in the past 24 h.

Generally, itch intensity measured with the numerical rating scale (NRS) can be categorized into no itch (NRS, 0), mild itch (NRS, 1–2), moderate itch (NRS, 3–6), severe itch (NRS, 7–8), and very severe itch (NRS, 9–10). Another category is also widely used as: no itch (NRS, 0), mild itch (NRS, 1–3), moderate itch (NRS, 4–6), and severe itch (NRS, 7–10). The PP‐NRS is a widely utilized instrument used in routine dermatological practices and clinical trials. The most appropriate threshold for defining a clinically relevant, within‐person response is reported to be a ≥2‐ to 4‐point change in the PP‐NRS.^8^

### 3.2 Visual analogue scale

The visual analogue scale (VAS) consists of a horizontal 10‐cm line with two end points of 0 (zero, no itch) at the left and 10 (the worst itch imaginable) at the right. Patients are instructed to rate their itch severity from zero to 10 by placing a mark on the line. The score is provided out of 10 (cm) or 100 (mm), e.g., 7.2 out of 10 (cm), or just 7.2/10, as provided in the NRS.

### 3.3 Verbal rating scale

The verbal rating scale (VRS) is a five‐point Likert scale comprising adjectives that describe various levels of itch intensity: 0 = no itch; 1 = mild itch; 2 = moderate itch; 3 = severe itch; and 4 = very severe itch. Patients are asked to rate their itch on average and at its worst in the past 24 h.

### 3.4 5‐D itch scale

The 5‐D itch scale is a brief and multidimensional questionnaire.^9^ This tool is designed to evaluate not only the itch intensity but also its impact on QOL. It comprises five dimensions: duration, degree, direction, disability, and distribution. The score ranges from 5 (no itch) to 25 (severe itch). The Japanese version of the 5‐D itch scale was translated by Ebata et al^10^ and is currently available.

### 3.5 Others

Other multidimensional itch measures include the Dermatology Life Quality Index (DLQI) and the ItchyQoL, both of which are capable of evaluating the QOL of patients with chronic itch. The DLQI is an assessment tool for QOL with 10 items, including an impact of itch on patients' daily lives. Although the DLQI is not itch‐specific, this tool is widely used for assessing QOL in patients with chronic itch^11^ and is effective for detecting the functional impact of QOL.

The ItchyQoL is an itch‐specific patient‐reported outcome measure designated for the assessment of the QOL in patients with chronic itch, irrespective of the presence or absence of visible skin lesions. It has 22 items and encompasses three domains: symptoms, functions, and emotions.^12^, ^13^

## 4 PATHOPHYSIOLOGY OF ITCH: ITCH PATHWAY FROM THE SKIN TO THE BRAIN

**FIGURE 1:**
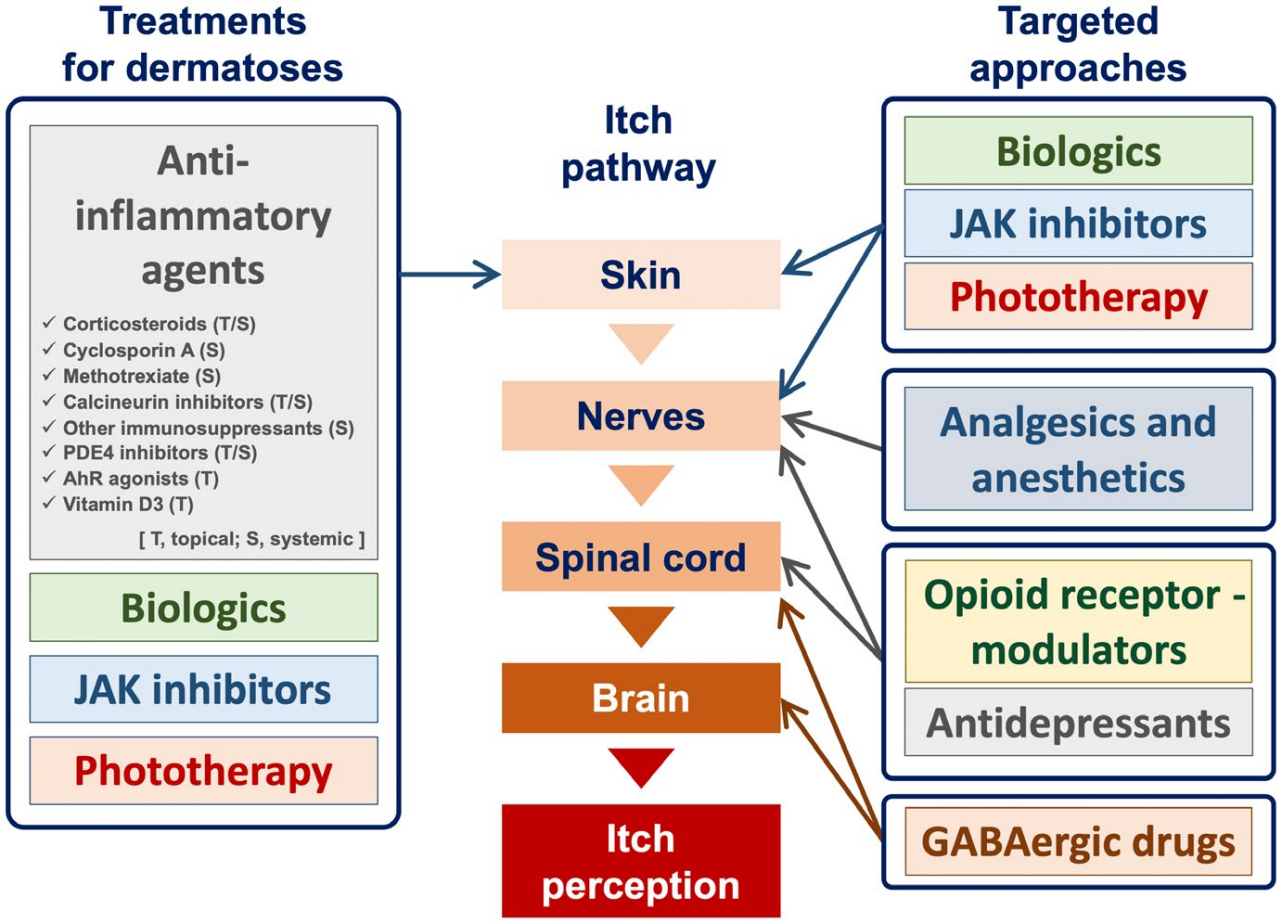
Itch pathway from the skin to the brain and targeted approaches for itch. The four consecutive steps of the skin, peripheral sensory nerve fibers, the spinal cord, and the brain are of importance in the sense of itching. A variety of treatment approaches targeting these four steps are currently in use in clinical practice. JAK, Janus kinase; PDE4, phosphodiesterase‐4.

The four consecutive steps of the skin, peripheral sensory nerves, the spinal cord, and the brain are essential in the sense of itching (Figure 1).

**FIGURE 2:**
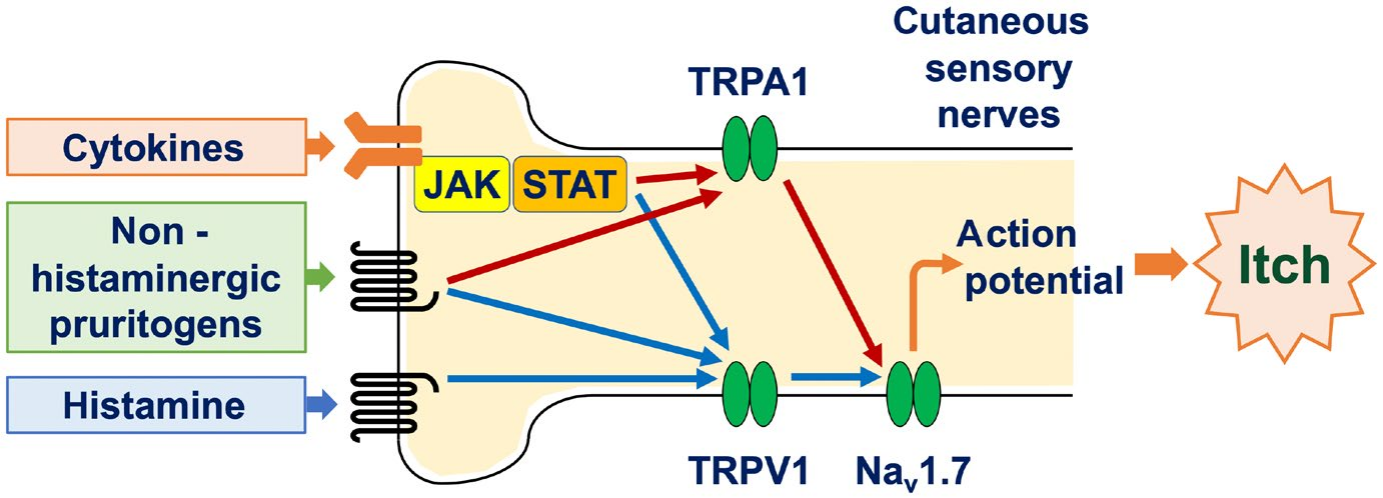
Itch transduction in cutaneous sensory nerve fibers. Pruritogens (substances that elicit itch) stimulate peripheral sensory nerve fibers in the skin via their receptors, thereby generating action potentials through the opening of ion channels, including transient receptor potential (TRP) A1 and V1 and voltage‐gated sodium channels (Na_v_), particularly Na_v_1.7. JAK, Janus kinase; STAT, signal transducer and activator of transcription.

Chemical itch typically develops when itch event(s) occur in the periphery. Such itch events encompass allergic skin inflammation (e.g., atopic dermatitis and prurigo nodularis) and external stimuli (e.g., insect bites). These itch events facilitate the release of pruritogens from various types of cells in the periphery. These pruritogens include histamine and other nonhistaminergic substances, including amines, neuropeptides, lipid mediators, and cytokines (particularly type 2 cytokines, e.g., interleukin [IL]‐4, IL‐13, and IL‐31) (Table 2). These pruritogens stimulate sensory nerve fibers in the skin via their receptors, thereby generating the electrical signals (action potential) through the opening of ion channels, including transient receptor potential A1 (TRPA1) and TRPV1, and voltage‐gated sodium channels (Na_v_), particularly Na_v_1.7 (Figure 2).^1^, ^14^

**FIGURE 3:**
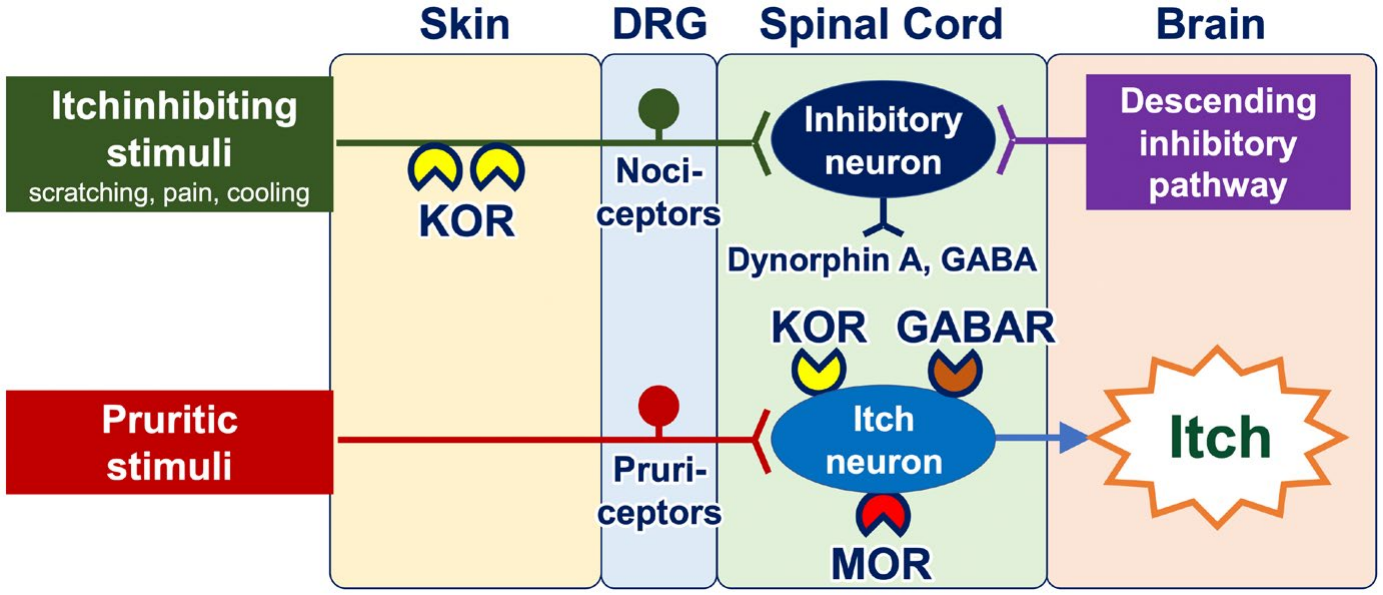
Itch neural circuit from the skin to the brain. Neural itch signals are transmitted from the skin to the brain. In the spinal cord, the transmission of itch signals is subject to modulation by the opioid and/or GABA systems. Interneurons express propruritic mu opioid receptors (MORs) and antipruritic kappa opioid receptors (KORs). MORs potentiate neural itch signals when stimulated by endogenous opiates (beta‐endorphin) or exogenous ligands such as morphine. KORs attenuate itch signals in response to dynorphin A and GABA from inhibitory interneurons. Inhibitory interneurons are positively regulated by both nociceptors from the periphery and the descending inhibitory pathway that originates from the brain. DRG, dorsal root ganglion; GABAR, GABA receptor.

Once transmitted into the spinal cord, neural itch signals are conveyed to interneurons and subsequently to projection neurons. The transmission of itch signals in the spinal cord is subject to modulation by the opioid and/or GABA systems. Interneurons express propruritic and antipruritic opioid receptors, namely mu opioid receptors (MORs) and kappa opioid receptors (KORs), respectively. MORs are activated by endogenous opiates (beta‐endorphin) or exogenous ligands such as morphine, which then potentiate neural itch signals. Conversely, KORs serve to attenuate itch signals when activated by dynorphin A and GABA from helix–loop–helix family member B5 (Bhlhb5)–expressing inhibitory interneurons.^15^ Bhlhb5‐expressing inhibitory interneurons are positively regulated by both nociceptors from the periphery (which are activated by scratching, cooling, and pain) and the descending inhibitory pathway arising from the periaqueductal gray matter in the brain (Figure 3). Additionally, glial cells (particularly astrocytes) exert a positive influence on itch‐conveying interneurons through a signal transducer and activator of transcription 3 (STAT3)–dependent manner.^16^

Neural itch signals are then conveyed toward the brain through the spinothalamic tract and the spinoparabrachial tract. In the brain, various regions receive itch signals that are projected. Regions that are highly selective for itch‐selective areas are postulated to exist in the precuneus and the posterior cingulate cortex.^17^ The precuneus is activated by itch stimuli but not painful sensations. Furthermore, this area is reportedly linked with happiness, indicating that itching may impair the patients' subjective happiness.^18^

Despite significant advancements in the understanding of chemical itch pathway, the mechanisms underlying mechanical itch remain largely elusive.^19^ Similarly, the neural processes underlying hyperknesis and alloknesis are yet to be fully elucidated.^15^

## 5 TREATMENT APPROACHES FOR ITCH

A variety of treatment approaches are currently in use in clinical practice, either as approved treatments or as off‐label uses (Figure 1).

### 5.1 Treatment approaches for pruritic dermatosis

When primary pruritic dermatosis is evident in patients presenting with itch, the initial step to reduce itch is to treat the dermatosis. In cases where dry skin is evident, the use of topical emollients or moisturizers can effectively alleviate the itching sensation.^20^, ^21^ Additionally, treatment approaches for pruritic dermatoses encompass a range of options, including topical/systemic anti‐inflammatory agents (e.g., corticosteroids and other immunosuppressants), biologics, Janus kinase (JAK) inhibitors, and phototherapy.^22^, ^23^, ^24^, ^25^, ^26^

### 5.2 Treatment approaches acting on the skin and peripheral sensory nerves

**FIGURE 4:**
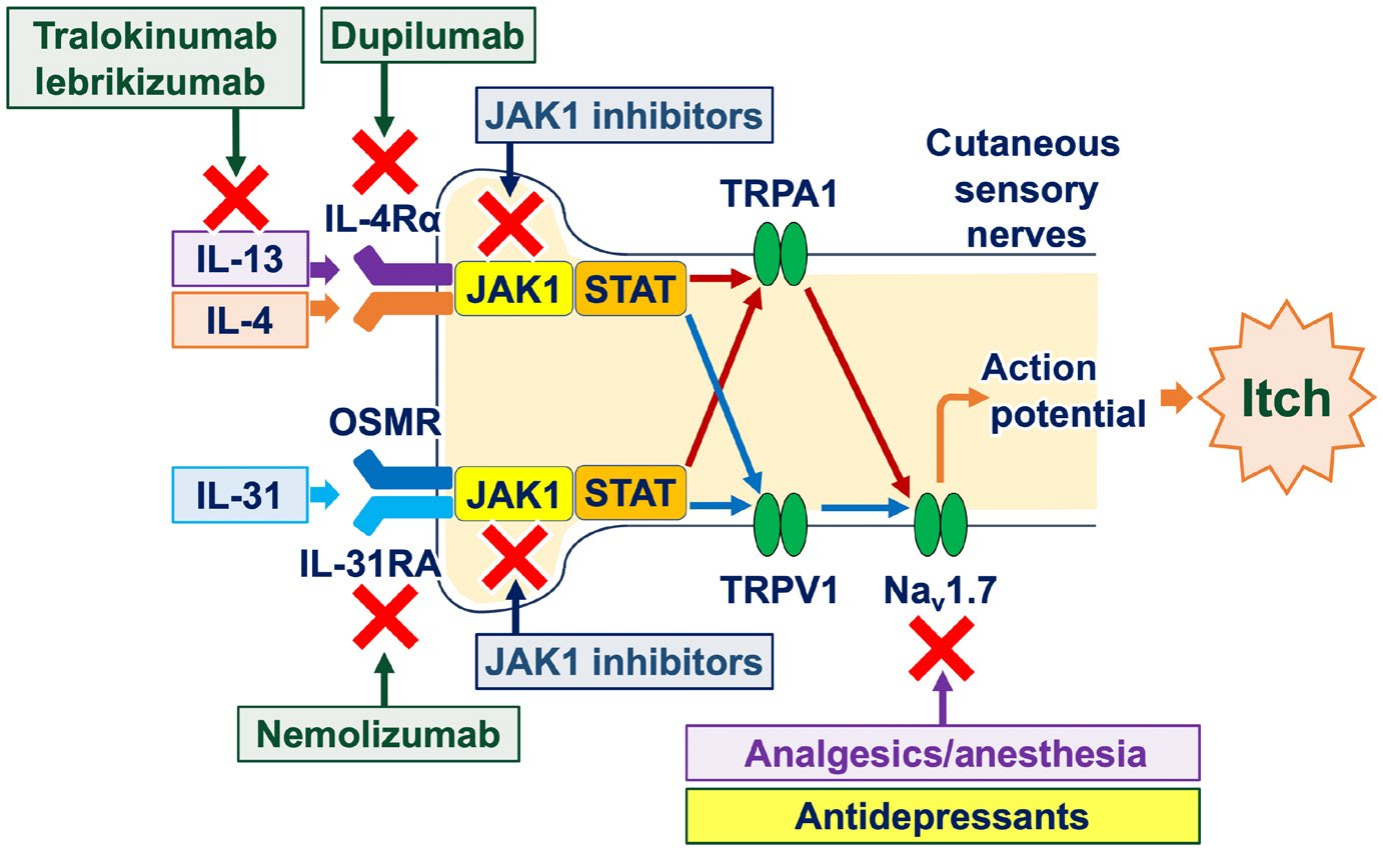
Mechanisms of action of targeted approaches for cutaneous pruritus in sensory nerve fibers. Biologics target cytokines or cytokine receptor components. Janus kinase 1 (JAK1) inhibitors regulate the generation of action potentials in nerve fibers. Analgesics/anesthesia and antidepressants act on voltage‐gated sodium channels (Na_v_). IL, interleukin; OSMR, oncostatin M receptor; STAT, signal transducer and activator of transcription; TRP, transient receptor potential.

In addition to their anti‐inflammatory effects, biologics and JAK inhibitors possess direct antipruritic properties. Peripheral sensory nerve fibers express receptors for type 2 cytokines, particularly IL‐4, IL‐13, and IL‐31.^27^ Upon activation of these cytokine receptors, JAK1 and STAT3 in peripheral sensory nerve fibers undergo phosphorylation, leading to the opening of ion channels (TRPA1, TRPV1, and Na_v_1.7) and the generation of action potentials. Therefore, biologics that target type 2 cytokine signaling pathways (e.g., nemolizumab, dupilumab, tralokinumab, and lebrikizumab) and small molecules that inhibit JAK1 phosphorylation (e.g., delgocitinib, ruxolitinib, abrocitinib, and baricitinib) are capable of inhibiting the generation of action potentials, resulting in the alleviation of itch (Figures 2 and 4).^28^, ^29^, ^30^

Phototherapy also possesses antipruritic properties besides anti‐inflammatory effects. Its mechanism of action involves the modulation of intraepidermal nerve fibers and epidermal opioid systems (i.e., MOR/KOR expression‐balance).^23^

### 5.3 Treatment approaches acting on peripheral sensory nerves

Antihistamines are well‐established antipruritic agents that inhibit H1R, which is expressed by itch‐transmitting peripheral sensory nerve fibers and thus attenuates histaminergic itch. Histaminergic itch is a significant contributor to itch observed in urticaria and insect bites/stings. These forms of itch can be effectively treated with antihistamines.^31^ However, the other majority of itching cases outside of these dermatoses may result from nonhistaminergic itch mechanisms, and, thus, antihistamines may not be the optimal treatment option.

Analgesics and anesthetics inhibit the activation of ion channels (particularly Na_v_ channels) and the generation of action potentials (Figure 4). Topical creams containing lidocaine are available as over‐the‐counter antipruritics in Japan and other countries. Topical ketamine (10% or 5%)–amitriptyline (5%)–lidocaine (5%) (TKAL) has been demonstrated to alleviate both inflammatory itch (itch in atopic dermatitis and prurigo nodularis) and neuropathic itch (brachioradial pruritus and other forms of neuropathic itch).^32^

The use of coolants (cooling agents) result in partial and transient alleviation of the itching sensation.^33^ They activate cold‐sensing TRPM8‐expressing nociceptors, which, in turn, stimulate Bhlhb‐5‐expressing itch‐inhibitory interneurons in the spinal cord (Figure 3). Topical formulations containing TRPM8 agonists menthol (1%–3%) and/or camphor are available over‐the‐counter in Japan and other countries. Of note, a subset of patients with chronic itch report exacerbation of their itch when exposed to cold^33^, ^34^ and they should be recommended to avoid cold sensation.^35^

Capsaicin, an active alkaloid compound contained in chili peppers and a ligand for TRPV1, has been demonstrated to alleviate itching when applied topically.^36^ The precise mechanism of action of capsaicin in the itch treatment remains to be elucidated. Treatment with capsaicin may result in functional and/or structural alterations of TRPV1‐expressing peripheral sensory nerve fibers that are involved in the transmission of itching sensations. The capsaicin 8% patch is indicated in adults for the treatment of neuropathic pain associated with diabetic peripheral neuropathy and postherpetic neuralgia in some countries, but it is not approved for itching conditions.^37^ Topical formulations containing capsaicin at lower concentrations are available over‐the‐counter in Japan and other countries.

### 5.4 Therapeutic approaches acting on peripheral nerves and the spinal cord

**FIGURE 5:**
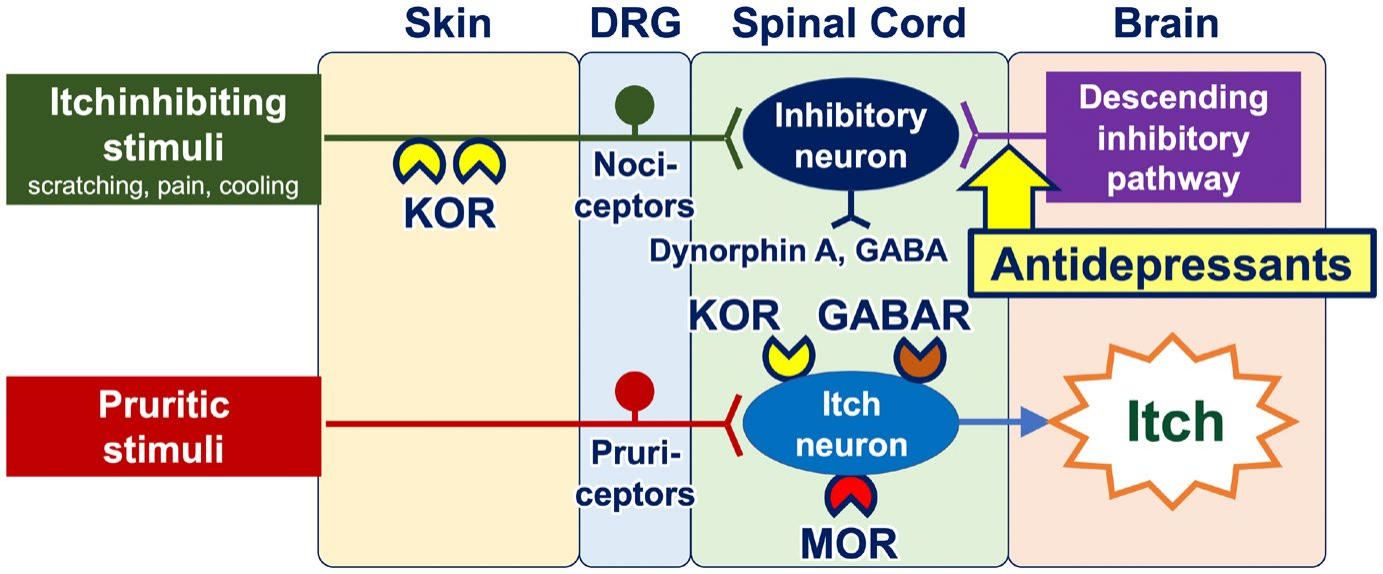
Mechanisms of antipruritic action of antidepressants in the central nervous system. Antidepressants activate the descending adrenergic itch‐inhibitory pathway in the spinal cord and the brain. Note, antidepressants also act on sodium channels on peripheral nerve fibers (see Figure 4). DRG, dorsal root ganglion; GABAR, GABA receptor; MOR, mu opioid receptor; KOR, kappa opioid receptor.

Antidepressants (e.g., mirtazapine and duloxetine) function as antipruritics.^38^ Their mechanisms of action include the modulation of peripheral nerve fibers via sodium channels (resulting in the blocking of action potentials) and the activation of the descending adrenergic itch‐inhibitory pathway in the spinal cord and the brain (Figures 4 and 5).^39^

**FIGURE 6:**
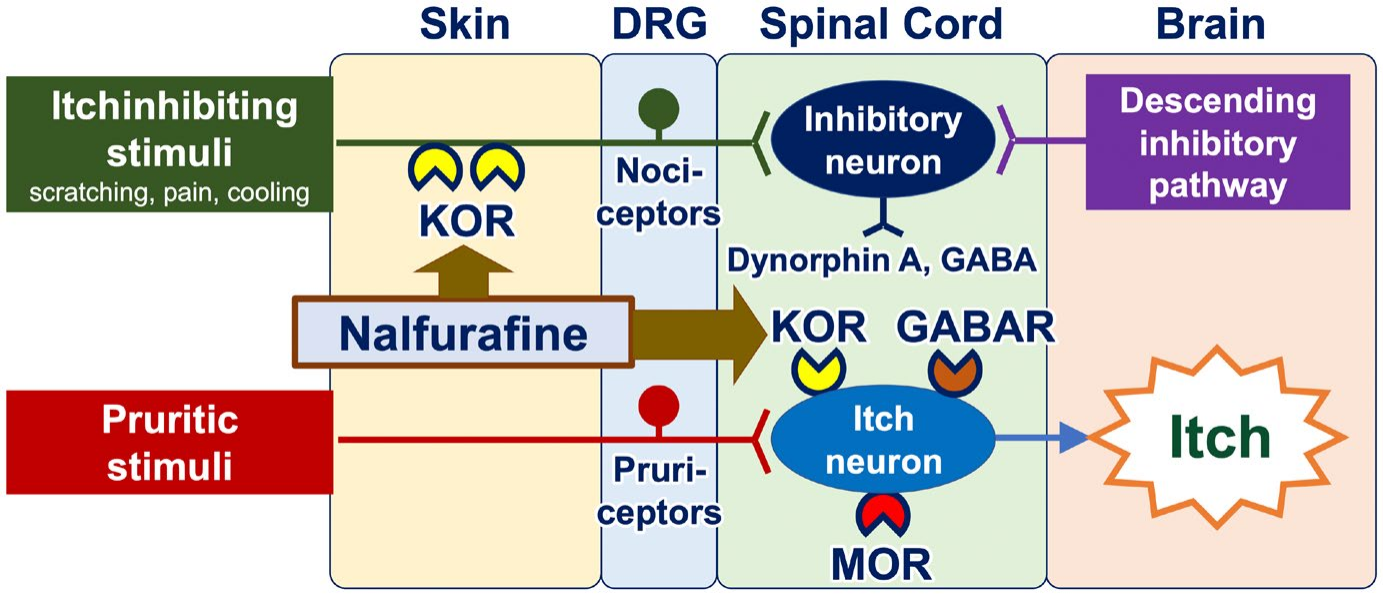
Mechanisms of action of opioid receptor modulators in the treatment of itch. Nalfurafine, an opioid receptor modulator approved in Japan for intractable itch in patients undergoing hemodialysis and those with chronic kidney disease, acts on antipruritic kappa opioid receptors (KORs) expressed by nociceptors in the periphery as well as interneurons in the spinal cord. Difelikefalin, another opioid receptor modulator, favorably activates peripheral KORs. DRG, dorsal root ganglion; GABAR, GABA receptor; MOR, mu opioid receptor.

Opioid receptor modulators have the potential to be employed as antipruritics. The upmodulation of antipruritic KORs and downmodulation of propruritic MORs appear to attenuate itch^40^, ^41^, ^42^ (Figure 6). Butorphanol (a nasal spray that functions as both a KOR agonist and an MOR antagonist), difelikefalin (an injectable KOR agonist), and nalfurafine (a KOR‐activating tablet) have been reported to be effective in the treatment of intractable itch, such as chronic kidney disease–associated pruritus (CKD‐aP), chronic liver disease–associated pruritus, notalgia paresthetica, and other inflammatory pruritic dermatoses (e.g., atopic dermatitis and prurigo nodularis).^43^, ^44^, ^45^, ^46^, ^47^

### 5.5 Therapeutic approaches acting on the spinal cord and the brain

**FIGURE 7:**
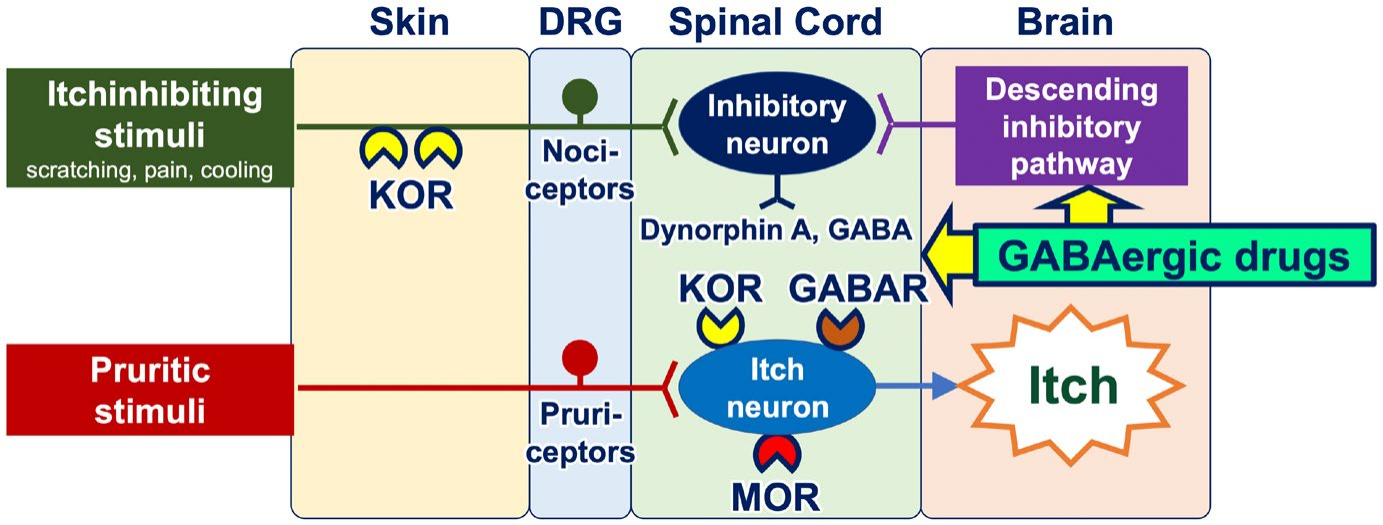
Mechanism of action of GABAergic drugs in the treatment of itch. GABAergic drugs have been demonstrated to downregulate itch‐conveying intermittent neurons in the spinal cord, as well as to activate the descending itch‐inhibitory pathway. DRG, dorsal root ganglion; GABAR, GABA receptor; MOR, mu opioid receptor; KOR, kappa opioid receptor.

GABAergic drugs (e.g., gabapentin, pregabalin, and mirogabalin) downregulate itch‐conveying intermittent neurons in the spinal cord, as well as activate the descending itch‐inhibitory pathway that originates in the brain and descends to the spinal cord (Figure 7). These agents have been shown to be particularly effective in the treatment of noninflammatory, neuropathic itch, including CKD‐aP, brachioradial pruritus, and notalgia paresthetica.^38^, ^48^

## 6 CUTANEOUS PRURITUS: ITCH WITH NO PRIMARY PRURITIC DERMATOSIS

Itch typically arises from inflamed skin, which may be the result of allergic inflammation or infectious skin (e.g., fungal, parasitic, and viral infections). On the other hand, a certain population of patients with itch present with uninflamed or normal‐appearing skin. These skin conditions may or may not result in secondary skin changes due to scratching. This itch condition is coined “cutaneous pruritus” in the Japanese Dermatological Association's guideline,^21^ “pruritus with no underlying dermatosis,” or “generalized pruritus without rash” if itch is generalized, in the British Association of Dermatologists' guidelines for the investigation and management of generalized pruritus in adults without an underlying dermatosis, 2018,^49^ and “pruritus on non‐diseased skin” in a position paper of the International Forum for the Study of Itch.^50^ The Japanese guideline further categorizes cutaneous pruritus into two phenotypes: generalized cutaneous pruritus and localized cutaneous pruritus. Generalized pruritus refers to presence of itch on a wide area of the body, while localized pruritus is defined as itch fixed and localized to a specific part of the body.^21^

Hereinafter, the term cutaneous pruritus is used to describe itch without primary pruritic dermatosis and observable primary rash.

## 7 CAUSES AND CHARACTERISTICS OF CUTANEOUS PRURITUS

**FIGURE 8:**
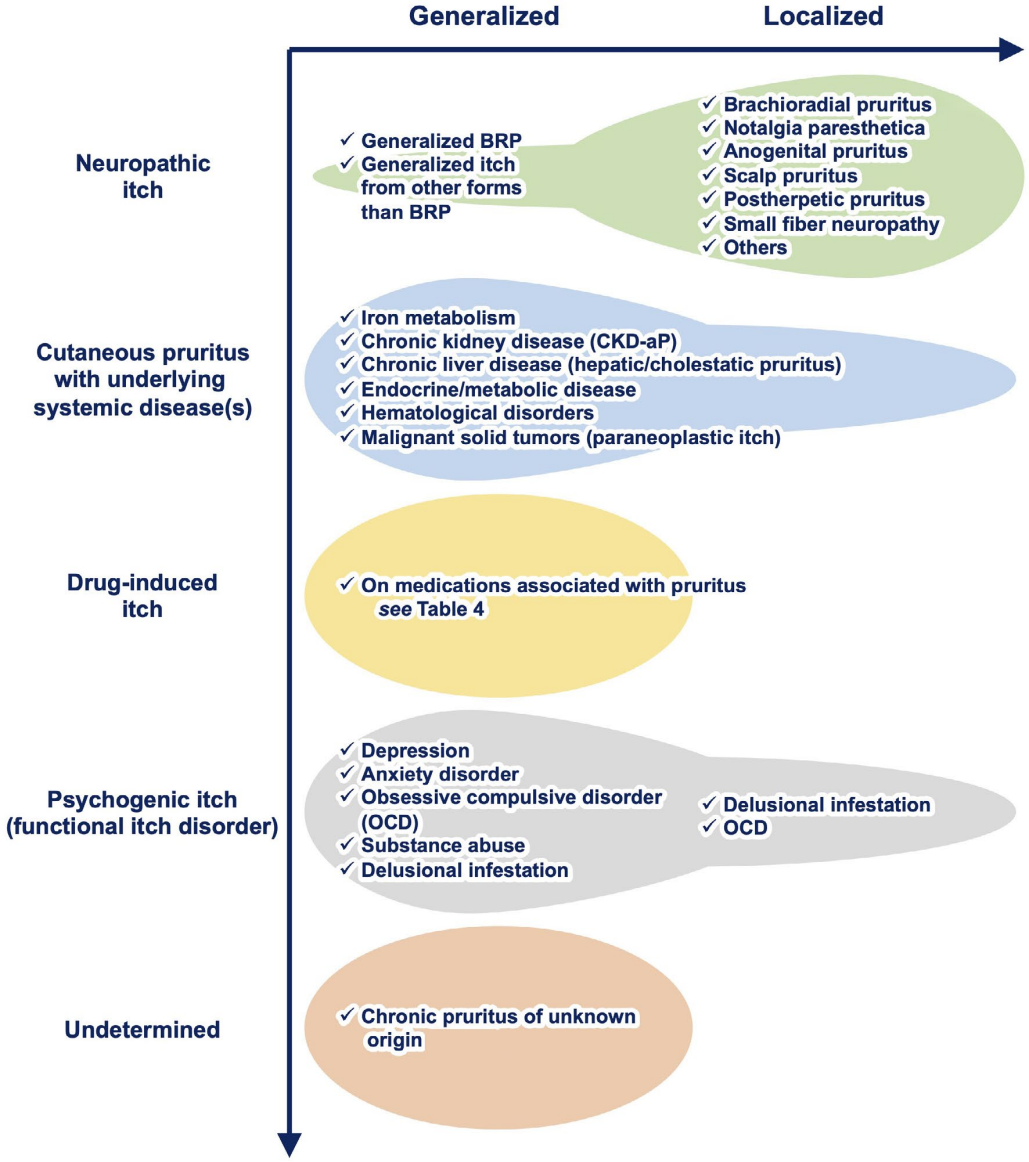
Causes of cutaneous pruritus. Cutaneous pruritus can be classified into two categories based on its distribution: Localized cutaneous pruritus and generalized cutaneous pruritus. Localized cutaneous pruritus is indicative of neuropathic cause, whereas generalized cutaneous pruritus suggests underlying systemic disease(s), drug‐induced itch, psychogenic itch (also known as functional itch disorder), or chronic pruritus of unknown origin (CPUO). BRP, brachioradial pruritus.

The causes of cutaneous pruritus are diverse, with some patients exhibiting a single cause, while others present with mixed causes (Figure 8).

### 7.1 Neuropathic itch

Neuropathic itch, also called “neurologic itch,” is primarily initiated, triggered, and/or caused by a lesion or disease to the somatosensory nervous system. There are many subtypes of neuropathic itch.

Neuropathic itch favors a localized or dermatomal distribution. Consequently, it is classified as localized cutaneous pruritus. However, in some cases, neuropathic itch can be generalized resulting from neural sensitization, in which limited nerve damage contributes to the increase in neurotransmitter release and hyperexcitable spinal neurons.^51^ Given that neuropathic itch has its origin in nerve damage, it could be characterized by a predominance of nociceptive symptoms (e.g., stinging burning, sunburn‐like sensation, pain, and hurting). However, a recent study has indicated that these nociceptive symptoms are more evident in inflammatory itch rather than neuropathic itch.^52^

The initial treatment approach for neuropathic itch is the removal of the underlying neuropathic cause (e.g., surgery for cervical hernia), if it is treatable. Other treatment approaches include the use of antidepressants and GABAergic drugs. Opioid receptor modulators are reported to alleviate itch in some subsets of neuropathic itch. Antihistamines are typically ineffective.

#### 7.1.1 Brachioradial pruritus

Brachioradial pruritus affects the dorsolateral upper extremities (corresponding to the C5/C6 dermatome and, on occasion, in between C6 and C8) and is most commonly observed in middle‐aged women. It is less prevalent in individuals with darker skin types when compared with those with lighter skin types (e.g., Fitzpatrick skin types I and II). Cervical radiculopathy and/or neuropathy may be the underlying cause of this condition. The exacerbating factors include ultraviolet radiation (i.e., sun exposure especially in warmer summer months). On the other hand, cold sensation may immediately relieve itching in brachioradial pruritus. This phenomenon is frequently designated to the ice pack sign. Brachioradial pruritus may manifest unilaterally or bilaterally,^53^ and sometimes it may be generalized.^54^ The diagnostic evaluation encompasses a through history with physical examinations (particularly the ice pack sign test) and imaging modalities (x‐rays, computed tomography, and magnetic resonance imaging) for detecting radiculopathy, if indicated.

#### 7.1.2 Notalgia paresthetica

Notalgia paresthetica refers to neuropathic itch localized to the unilateral medial border of the inferior scapula, but it can sometimes be bilateral. This type of itch is frequently observed in middle‐aged and older women, but it can also be seen in males with no racial predilection. The underlying mechanism is the impingement or injury of the T2 to T6 nerves, which can be attributed to muscle spasms and/or degenerative vertebral changes and herniated intravertebral discs. The dorsal rami of these nerves are at increased risk of trauma/entrapment because of their angular 90‐degree course through the muscles.^55^ The diagnostic workup includes a through history and targeted radiologic examination. The ice pack sign may also be a good indicator in patients with notalgia paresthetica, although it is not a definitive diagnostic tool.^56^ A recent clinical study reported a significant improvement of itch by the KOR agonist difelikefalin.^47^

#### 7.1.3 Anogenital pruritus

Anogenital pruritus is defined as itch localized to the anal, perianal, or genital skin. This type of itch is four times more prevalent in middle‐aged men than women.^57^ The underlying causes for anogenital pruritus remain largely unclear; however, degenerative changes to the lower spine (between L4 and S2) resulting in compression of a nerve or nerve root may contribute to its development. Additionally, postherpetic neuralgia and diabetic neuropathy have been identified as potential causative factors for anogenital pruritus.^58^, ^59^

#### 7.1.4 Scalp pruritus

Scalp pruritus is thought to primarily arise from aberrant function of the trigeminal nerves.^60^ There is evidence to suggest that the skin microbiome play a role in exacerbating itch.^61^ A similar condition is also referred to as scalp dysesthesia (burning scalp syndrome), which presents burning and tingling and sometimes an itching sensation.^62^, ^63^ The question of whether scalp pruritus and scalp dysesthesia are on the same spectrum remains unanswered.^51^

#### 7.1.5 Postherpetic pruritus

Postherpetic pruritus (postherpetic itch) is a neuropathic itch localized on the area(s) previously affected by herpes zoster.^64^ The itch is more prevalent on the head, neck, and face (regions innervated by the trigeminal nerve), followed by the trunk.^65^, ^66^ The causes include a reduction in intraepidermal nerve fiber densities representing small fiber neuropathy.^67^, ^68^ Of note, postherpetic pruritus not only causes itch itself but also itch sensitization phenomena (increased sensitivity to touch‐evoked itch [punctate hyperknesis]). This itch and punctate hyperkenesis improved, accompanied by restored intraepidermal nerve fiber densities in the affected area.^68^

#### 7.1.6 Small fiber neuropathy

Small fiber neuropathy (SFN) is a not uncommon disorder that affects the thinly myelinated A‐δ and unmyelinated C fibers, both of which are involved in itch conduction in the skin. SFNs are often idiopathic yet may result from a multitude of underlying conditions, including diabetes, chronic kidney disease, infectious diseases (e.g., HIV, hepatitis C infection, and Chagas disease), autoimmune diseases (e.g., Sjögren syndrome), paraneoplastic syndromes, and genetic diseases (e.g., Fabry disease and familial amyloid polyneuropathy).^69^, ^70^, ^71^ The symptoms of SFN encompass autonomic symptoms (e.g., dry eyes/mouses, constipation, bladder incontinence, and dizziness), as well as sensory symptoms (e.g., pain, itch burning, tingling, or numbness). This dysesthesia typically starts distally, affecting the limbs in a distal to proximal gradient, and may generalize. The diagnostic workup includes quantitative sensory testing and skin biopsy for the examination of intraepidermal nerve fiber densities, which are reduced in SFN.^72^, ^73^

#### 7.1.7 Others

Neuropathic itch may develop in association with syndrome(s) affecting the central nervous system, including poststroke pruritus, brain/spinal tumors, Creutzfeldt‐Jakob disease, trigeminal trophic syndrome, multiple sclerosis, and postsurgical.

### 7.2 Cutaneous pruritus with underlying systemic disease(s)

A variety of systemic diseases are associated with cutaneous pruritus.^74^

#### 7.2.1 Disorders of iron metabolism

Both iron deficiency and iron overload can be associated with generalized pruritus, but, in some cases, they may also cause localized itch rather than generalized itch. For example, localized vulvar itch may be indicative of iron deficiency.^75^ The precise mechanism underlying itch associated with abnormal iron metabolism remains unclear.

Iron deficiency is frequently attributable to particular diet habits (e.g., vegetarian and vegan), gastrointestinal bleeding, menstrual blood loss, pregnancy, and blood loss from hemorrhoids. In patients with iron deficiency–associated generalized pruritus, the itching sensation can be attenuated or, in some cases, completely diminished, shortly after the commencement of iron replacement therapy.^76^ Iron overload such as hemochromatosis also elicit pruritus, and treatment for iron overload with venesection or iron chelation can result in attenuation of itching sensation.^77^

#### 7.2.2 Chronic kidney disease

CKD‐aP, formerly known as uremic pruritus, affects approximately 60% of patients on hemodialysis as well as patients with stage 3 to 5 CKD. Despite this high prevalence, CKD‐aP is frequently underdiagnosed and untreated. In DOPPS (Dialysis Outcomes and Practice Patterns Study), the largest epidemiologic study with more than 35 000 patients undergoing hemodialysis, 18% of patients on hemodialysis exhibiting CKD‐aP used no treatment for pruritus and 17% did not report their itch to healthcare staff. Furthermore, 69% of the medical staffs underestimated the prevalence of CKD‐aP in their institutions.^78^

The most commonly affected areas of CKD‐aP are the back and limbs, even though more than 50% of patients complain of generalized pruritus. CKD‐aP is more prevalent in patients undergoing hemodialysis than in those undergoing peritoneal dialysis. It is noteworthy that pruritus is associated with a high prevalence of depression mood as well as an increased mortality risk, which may be partially attributable to cardiovascular disorders from sleep disturbances.^2^, ^78^, ^79^, ^80^

The pathogenesis of CKD‐aP appears to be complex, with a multitude of factors involved, including uremic toxins, imbalance in the endogenous opioid system, neuropathy, and others.

A recent study from Japan demonstrated that protein‐bound uremic toxins, which are difficult to remove with hemodialysis, play a significant role in itch in patients undergoing hemodialysis. The serum level of protein‐bound uremic toxins (indoxyl sulfate, p‐cresyl sulfate, indole acetic acid, phenyl sulfate, and hippuric acid) have been found to be associated with the presence of pruritus (odds ratio, 1.65; *p* = 0.027).^79^ On the other hand, DOPPS revealed that serum levels of calcium, phosphate, calcium‐phosphate products, and parathyroid hormone do not correlate with itch severity in patients undergoing hemodialysis. Kt/V (a measure of the efficacy of a hemodialysis session) lower than 1.5 is associated with pruritus; however, the correlation between Kt/V and itch severity is not established.^78^

An imbalance in the endogenous opioid system is also an important player in itch in hemodialysis patients, who exhibit increased MOR activity and decreased KOR activity. KOR agonists (nalfurafine and difelikefalin) have been reported to reduce itch severity in patients on hemodialysis in clinical trials.^44^, ^81^ Furthermore, difelikefalin has been shown to improve CKD‐aP in nondialysis‐dependent patients.^82^

Microinflammation in the skin may contribute to CKD‐aP. Serum C‐reactive protein levels and serum IL‐2 levels are correlated with itch severity in hemodialysis patients.^78^ Uremic neuropathy and paresthesia, which includes restless leg syndrome, have been reported to be associated with CKD‐aP.^83^ Functional and structural alterations in the brain, as revealed through functional magnetic resonance imaging studies, have also been reported to be associated with CKD‐aP.^84^

The treatment approaches for CKD‐aP include topical emollients, opioid modulators, phototherapy, antidepressants, and GABAergic drugs. Dry skin is a common occurrence in patients undergoing hemodialysis, and it is often accompanied by pruritus. Topical emollients provide at least partial relief from hemodialysis‐associated itch. Opioid modulators not only act on the endogenous opioid system but also improve microinflammation in the periphery.^44^, ^81^ Phototherapy also targets microinflammation in the skin. GABAergic drugs and antidepressants modulate neuronal activities in both the peripheral and central nervous systems.

#### 7.2.3 Chronic liver disease (especially cholestasis)

The close association between itch and jaundice has been recognized since antiquity and the ancient Greek physician Aretaeus (Ἀρεταῖος) of Cappadocia described cholestatic itch in the second century ad.^85^ Itch associated with chronic liver disease is referred to as hepatic pruritus. When cholestasis appears to significantly contribute to hepatic pruritus, it is also referred to as cholestatic pruritus. The list of chronic liver diseases that may cause hepatic pruritus/cholestatic include not only cholestatic diseases (e.g., primary biliary cirrhosis, primary sclerosing cholangitis, intrahepatic cholestasis of pregnancy, or hereditary pediatric cholestatic disorders) but also chronic liver diseases (e.g., chronic hepatitis C virus infection and nonalcoholic fatty liver disease).^86^ A large‐scale surveillance study conducted in Japan revealed that 40.3% of patients with chronic liver diseases complain of itch. The prevalence of itch varies among chronic liver diseases, with higher prevalences observed in overlap syndrome (60.0%), primary biliary cirrhosis (51.4%), chronic hepatitis C virus infection (45.4%), and nonalcoholic fatty liver disease (44.7%) and relatively lower prevalences in alcoholic liver disease (24.3%) and inactive hepatitis B virus carrier (22.2%). Cholestatic pruritus typically affects the limbs and the back, although it can be generalized.^87^, ^88^ Of note, hepatic pruritus can precede the appearance of jaundice.^89^

The pathogenesis of hepatic itch remains unclear. Recent studies have indicated that abnormalities in lysophosphatidic acid (LPA) and the endogenous opioid system play a role in the pathogenesis of cholestatic pruritus. The serum levels of LPA and the activity of its synthetic enzyme autotaxin are elevated in cholestatic patients and correlate with the severity of itch. LPA is reported to activate itch‐related sensory nerve fibers through its receptor LPAR5, and biliary drainage has been shown to reduce autotaxin activity and improve itching.^90^ Cholestatic patients exhibit increased serum levels of endogenous opioids, particularly MOR agonists. MOR agonists possess the propruritic properties, which may contribute to the sensitization and modulation of nervous systems, rather than directly activating itch‐associated sensory nerve fibers.^91^ However, the direct correlation between blood endogenous opioid levels and itch severity has yet to be demonstrated.^92^

The precise role of bile acids and bilirubin in the pathogenesis of hepatic pruritus remains uncertain. Serum levels of bile acids and bilirubin are not necessarily correlated with itch intensity in cholestatic patients. However, recent studies have demonstrated that these substances act as ligands for Mas‐related G‐coupled receptor X4, which is expressed by itch‐associated sensory nerves. Mouse studies have indicated that intradermal injection of bile acids induces scratching behavior, and that bilirubin levels in the skin but not in the serum correlate with scratching behavior.^93^, ^94^

The first‐line treatment for hepatic/cholestatic pruritus in Japan is nalfurafine hydrochloride, a KOR agonist tablet approved by the Pharmaceuticals and Medical Devices Agency. A shorter pruritus period and milder disease stage with less‐advanced fibrosis are reportedly associated with a favorable response to this drug.^95^, ^96^ Other opioid modulators, such as butorphanol nasal spray, are also reported to attenuate cholestatic pruritus.^97^

Biliary drainage is capable of reducing itch as well as improving jaundice. Additionally, intestinal bile acid transporter inhibitors and cholestyramine are also reported to improve cholestatic itch by decreasing the bile acid pool.^98^, ^99^ Rifampicin, which regulates autotaxin expression, and bezafibrate, whose antipruritic mechanism remains to be elucidated, have antipruritic properties in cholestasis. Similarly, phototherapy has been shown to be effective in treating cholestatic pruritus. The efficacies of antidepressants and GABAergic drugs for the treatment of cholestatic pruritus remain uncertain.^89^ Several studies have indicated that GABAergic drugs may worsen hepatic pruritus.^100^ Ursodeoxycholic acid and antihistamines are usually not effective.

#### 7.2.4 Endocrine/metabolic diseases

Patients with diabetes, particularly those with diabetic neuropathy, often complain of itching. Diabetes‐associated pruritus is typically localized to the scalp, trunk, ankles, feet, and genitals, but it can be generalized. The pathogenesis of diabetic itch may involve dysesthesia from diabetic neuropathy and anhidrosis‐related dry skin resulting from autonomic nerve fiber dysfunction. Potential treatment approaches include topical emollients for dry skin and, of course, glycemic control. Other medications, such as GABAergic drugs and antidepressants, might be beneficial, although their evidence base is limited.^101^

Thyroid dysfunction and parathyroid dysfunction may be associated with generalized pruritus, but there is a paucity of evidence from clinical studies to support an association between thyroid and parathyroid dysfunction and generalized pruritus.

#### 7.2.5 Hematological disorders

Generalized itch is one of the presenting symptoms associated with myeloproliferative neoplasms (MPNs), especially polycythemia vera (PV), essential thrombocytosis, and primary myelofibrosis. It is noteworthy that patients with myeloma do not typically present with generalized pruritus.^102^ Patients with PV and other forms of MPNs frequently report a characteristic and distinct form of pruritus, known as aquagenic pruritus.^103^ Aquagenic pruritus is defined as an intense itching sensation within a few minutes of the exposure to water (particularly hot water rather than cold water), without any apparent skin changes. The frequent itching sites of aquagenic pruritus are the upper and lower extremities. It is estimated that up to 70% of patients with PV experience aquagenic pruritus, and the onset of aquagenic pruritus often precedes the diagnosis of MPNs. The precise pathogenesis of MPN‐associated itch and aquagenic pruritus remains unclear, but a genetic mutation, basophils, and mast cells may contribute to these phenomena.^104^, ^105^, ^106^, ^107^ The JAK2 gain‐of‐function mutation (JAK2V617F) is present in up to 97% of patients with PV and in more than 50% of patients with other forms of MPNs. This mutation is closely associated with itch in MPNs, since the mutation burden is reportedly correlated with the occurrence of aquagenic pruritus.^108^ The JAK1/2 inhibitor ruxolitinib has been demonstrated to rapidly improve itch in MPNs.^105^ Treatment approaches for itch in PV include antidepressants^109^ and phototherapy.^110^, ^111^ Antihistamines are shown to be ineffective in improving itch in MPNs.^112^ Itch in PV may persist even after the normalization of blood cell counts with venesection.^113^

Lymphoma (both Hodgkin and non–Hodgkin lymphoma) is also associated with generalized pruritus, with a prevalence of approximately 15% and 25%, respectively.^114^ In patients with Hodgkin lymphoma, itch severity is reported to be correlated with advanced disease stage and may serve as an indicator of poor diagnosis.^115^ The pathogenesis of lymphoma‐associated itch remains unclear. IL‐31 and thymic stromal lymphopoietin, which are possibly secreted by tumor cells and infiltrating immune cells, are reported to be involved in itch in Hodgkin lymphoma.^116^ The curative treatment of lymphoma has been shown to resolve lymphoma‐associated itch. The available literature reports various treatment options including oral corticosteroids, cimetidine, GABAergic drugs, antidepressants, and phototherapy.^49^

#### 7.2.6 Malignant solid tumors

Cutaneous pruritus in patients with solid malignant tumors, which is sometimes referred to as paraneoplastic pruritus, is a relatively rare phenomenon whose pathogenesis remains unclear. Some substances from tumor cells such as IL‐31, paraneoplastic neuropathy, and cancer treatments including radiotherapy may cause itching. When no neuropathic and systemic underlying diseases other than malignant tumors are detected, paraneoplastic pruritus should be considered as a potential diagnosis. Treatments for this itch include antidepressants and removal of tumors.^49^

### 7.3 Cutaneous pruritus as an adverse drug reaction (drug‐induced pruritus)

Itching has been reported as an adverse drug reaction of various drugs, including opioids and antibiotics (Table 4). This itch is usually generalized and infrequently localized. The precise mechanism underlying drug‐induced pruritus remains unclear but may vary depending on a drug/medication a patient is taking. Some drugs (e.g., chloroquine) act as pruritogens, directly activating sensory nerve fibers, while others (e.g., opiates and dipeptidyl peptidase 4‐inhibitors) involve opioid systems.^117^ The diagnosis of drug‐induced pruritus is difficult. When the itching is relieved by the cessation of the culprit drug(s) and recurs upon readministration of these drug(s), the diagnosis of drug‐induced pruritus can be made.

**TABLE 4:** Medications that may cause drug‐induced pruritus.

Group of drugs	Examples
Opioids and analgesics/anesthesia	Morphine, heroin, codeine, cocaine, fenoprofen, aspirin, other nonsteroidal anti‐inflammatory drugs, gold preparations
Antimicrobials and antibiotics	Chloroquine, β‐lactam antibiotics, rifampicin, polymyxin B
Cardiovascular medicines	Captopril, enalapril, clonidine, amiodarone, dopamine, quinidine, digitalis preparations, furosemide, hydrochlorothiazide
Metabolic medicines	Dipeptidyl peptidase 4 inhibitors, metformin, gliclazide, alloprinol
Neuroleptic and psychotropic drugs	Benzodiazepines, meprobamate, carbamazepine, imipramine, barbital
Steroids and hormones	Progesterone, estrogen, oral contraceptives, dexamethasone
Chemotherapeutics	Bleomycin
Other drugs	Hydroxyethyl starch, etretinate, radiopaque contrast agents

### 7.4 Psychogenic cause

It is estimated that at least 32% of patients with a psychiatric diagnosis complain of itch.^118^ Psychiatric disorders associated with cutaneous pruritus include depression, anxiety disorder, obsessive‐compulsive disorder, substance abuse, and delusional infestation. In the absence of a discernible somatic cause even after meticulous examinations for cutaneous pruritus, and when the itching sensation is attributed to a psychiatric disorder (and alleviated by psychotropic drugs and/or psychotherapies when psychiatric approaches are pursued), this itch can be diagnosed as psychogenic itch (also coined functional itch disorder).^119^ However, it is imperative that physicians refrain from mislabeling patients with cutaneous pruritus as having “psychogenic” itch.

### 7.5 Unknown origin

In instances where patients present with chronic itch and no underlying origin for pruritus can be determined despite a comprehensive and careful diagnostic workup, a diagnosis of chronic pruritus of unknown origin (CPUO) can be assigned.^120^, ^121^ CPUO often features “generalized” itch, but the extent to which this condition is generalized varies. It is estimated that approximately 6% of patients with chronic pruritus can be diagnosed as having CPUO.^122^ This itch may occur at any age. The pathogenesis of CPUO remains naturally uncertain. Some CPUO patients show immunologic profile skewed toward type 2 immunity, evidenced by peripheral eosinophilia^123^ and type 2–related lesional gene expressions.^124^, ^125^ Treatment agents targeting type 2 inflammation have been shown to improve itch in CPUO, including JAK inhibitors^126^ and dupilumab.^127^ A recent study has reported that downregulation of metabolic pathways related to catecholamine biosynthesis and tryptophan biosynthesis is observed in patients with CPUO.^128^ Further studies are required to elucidate this issue.

## 8 DIAGNOSTIC PROCEDURES FOR CUTANEOUS PRURITUS

**FIGURE 9:**
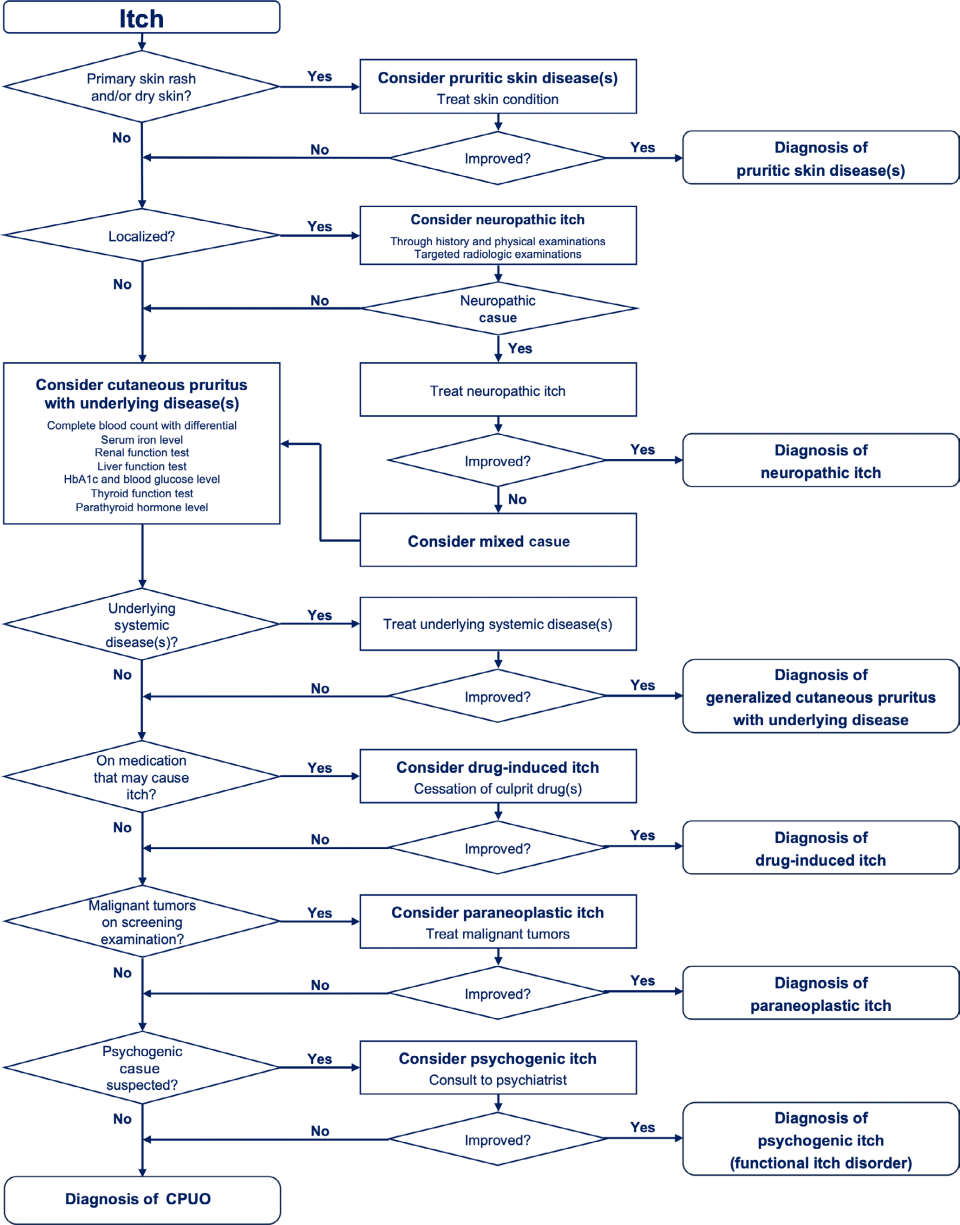
Diagnostic procedures for cutaneous pruritus. The initial step is to ascertain whether presence of primary rash is present on the skin. In the absence of pruritic rash, a diagnosis of cutaneous pruritus can be made, and further examination determines which type of cutaneous pruritus the patient has. CKD‐aP, chronic kidney disease–associated pruritus.

The diagnostic procedures for cutaneous pruritus are shown in Figure 9. The initial step is to ascertain that the presence of primary rash is apparent on the skin. In the absence of pruritic rash, a diagnosis of cutaneous pruritus can be made. In cases where a primary pruritic dermatosis is evident but the appropriate treatment for it does not alleviate the itch, then the possibility of a coexistence of a pruritic dermatosis with cutaneous pruritus should be considered.

Localized itch is favorably indicative of a neuropathic origin, whereas generalized itch is suggestive of alternative forms of cutaneous pruritus, including cutaneous pruritus with underlying systemic disease(s), drug‐induced itch, psychogenic itch (functional itch disorder), and CPUO. A comprehensive examination for underlying systemic disease(s) is essential for accurate diagnosis and appropriate treatment.

## FUNDING INFORMATION

The authors received no specific funding for this article.

## CONFLICT OF INTEREST STATEMENT

Nothing to declare.
